# Utilizing Gut Microbiota to Improve Hepatobiliary Tumor Treatments: Recent Advances

**DOI:** 10.3389/fonc.2022.924696

**Published:** 2022-07-18

**Authors:** Hao Qin, Baowen Yuan, Wei Huang, Yan Wang

**Affiliations:** ^1^ Key Laboratory of Cancer and Microbiome, State Key Laboratory of Molecular Oncology, National Cancer Center/National Clinical Research Center for Cancer/Cancer Hospital, Chinese Academy of Medical Sciences and Peking Union Medical College, Beijing, China; ^2^ Beijing Key Laboratory of Cancer Invasion and Metastasis Research, Department of Biochemistry and Molecular Biology, School of Basic Medical Sciences, Capital Medical University, Beijing, China

**Keywords:** hepatobiliary tumors, gut microbiota, metabolites, probiotics, antibiotics, fecal microbial transplantation

## Abstract

Hepatobiliary tumors, which include cholangiocarcinoma, hepatocellular carcinoma (HCC), and gallbladder cancer, are common cancers that have high morbidity and mortality rates and poor survival outcomes. In humans, the microbiota is comprised of symbiotic microbial cells (10-100 trillion) that belong to the bacterial ecosystem mainly residing in the gut. The gut microbiota is a complicated group that can largely be found in the intestine and has a dual role in cancer occurrence and progression. Previous research has focused on the crucial functions of the intestinal microflora as the main pathophysiological mechanism in HCC development. Intestinal bacteria produce a broad range of metabolites that exhibit a variety of pro- and anticarcinogenic effects on HCC. Therefore, probiotic alteration of the gut microflora could promote gut flora balance and help prevent the occurrence of HCC. Recent evidence from clinical and translational studies suggests that fecal microbiota transplant is one of the most successful therapies to correct intestinal bacterial imbalance. We review the literature describing the effects and mechanisms of the microbiome in the gut in the context of HCC, including gut bacterial metabolites, probiotics, antibiotics, and the transplantation of fecal microbiota, and discuss the potential influence of the microbiome environment on cholangiocarcinoma and gallbladder cancer. Our findings are expected to reveal therapeutic targets for the prevention of hepatobiliary tumors, and the development of clinical treatment strategies, by emphasizing the function of the gut microbiota.

## Introduction

Hepatobiliary tumors are malignant space-occupying lesions that occur in hepatocytes, the gallbladder, and intrahepatic bile ducts. They are associated with a poor survival rate and an aggressive phenotype (1). Primary liver cancer is the second leading cause of cancer-related death worldwide. Approximately 75% of patients with primary liver cancer have hepatocellular carcinoma (HCC) (2), and it was estimated that, in 2030, more than one million patients will die from liver cancer (3). The major risk factors for HCC are alcohol intake, viral hepatitis, and nonalcoholic steatohepatitis (NASH), which occurs in chronic liver disease patients (4, 5). HCC is invasive and has a high level of metastasis; however, as it is asymptomatic in its early stages, it is frequently diagnosed by imaging at an advanced stage (6). Serum α-fetoprotein is commonly used as a screening biomarker for patients with HCC in developing countries (7, 8). However, there is controversy regarding the use of α-fetoprotein due to its poor specificity and sensitivity, limiting its application in clinical practice (9). There is thus an urgent need to investigate and identify combinations of tumor markers that have a high level of prediction.

The human body consists of trillions of microbes that mainly live in the digestive system, and these include bacteria, viruses, fungi, and archaea (10). The ratio of microbial cells to human cells is roughly equal and the total bacterial count in the colon, the organ with the most microorganisms, is approximately 3.8 × 10^13^ (11). The human gut microbiota has a close relationship with the homeostasis and health maintenance of the host (12, 13). Many probiotics exhibit an antitumor activity through apoptotic signaling pathway activation or immune response enhancement, and they can help improve intestinal health and maintain intestinal flora (14). At the same time, probiotic metabolites may regulate various cellular and molecular processes and the composition of the gut microbiome (15), which plays a critical role in cancer prevention and supports anticancer therapies. Bacterial fermentation of carbohydrates and proteins produces a range of metabolites that have been shown to influence human health and are associated with many diseases either directly or indirectly (16). These metabolites interact with numerous important targets in several metabolic pathways and the molecular processes of malignant tumors.

The largest proportion of bacteria is located in the colon and direct bacterial actions and metabolites promote colorectal cancer initiation and development (17). The hepatic portal vein transports venous blood to the liver, which is subjected continually to bacterial products derived from the gut (18). The gut-liver axis forms a reciprocal interaction between the intestine and the liver that involves the intestinal microbiome and hepatic immune system, which mediates hepatobiliary disorders (19–21). An increasing number of studies have recently shown that the microbiota of the gut, intestinal commensal bacterial species, and their metabolites and products, have a vital function in the pathogenesis of or protection against the development of HCC (22–24). Antibiotic treatments are connected to decreased commensal intestinal bacterial diversity, which, in combination with anticancer therapies, affects the outcome of patients with HCC (25, 26). The promising strategy of fecal microbiota transplantation (FMT) for improving the immune response against cancer has been examined (27), and the enhanced effectiveness of inhibitors of the immune checkpoints with FMT may be a potential strategy to prevent HCC.

Here, we summarize the current progress in our comprehension of the gut microbiota in the treatment of hepatobiliary tumors, including HCC, cholangiocarcinoma (CCA), and gallbladder cancer (GBC). The fundamental mechanisms of the effects of metabolites derived from the gut microbiota on the occurrence of HCC are also discussed. These potential effective therapeutic or preventive strategies, including probiotics, antibiotics, and FMT, may lead to new noninvasive and cost-effective approaches for preventing and treating HCC in the future.

## Bacterial Metabolism and HCC Promotion

The gut-liver axis is an anatomical and physiological connection between the liver and gut. The gut microbiota, including metabolites, helps to maintain normal liver functions. Exposure of the liver with chronic lesions to bacterial metabolites results in liver damage and ultimately to the development of HCC (28). Synthesis of cholic acid, primary bile acids (PBAs), and chenodeoxycholic acid takes place *via* liver cholesterol, and several studies have examined the function of bile acid metabolism disorders in relation to the growth of HCC. Bile acids are categorized as PBAs and secondary bile acids (SBAs). SBAs are derived from PBAs that are converted by gut bacteria and microbial bile acid products and could, thus, be promoters of liver cancer (29). The SBA deoxycholic acid (DCA) is a byproduct of intestinal bacterial metabolism that promotes hepatocarcinogenesis, and the reduction of DCA levels prevents the formation of liver cancer in obese mice (30). The STHD-01 model of steatohepatitis induced by high-fat diet (HFD), which promotes HCC without the administration of chemical carcinogens, shows that the hepatic accumulation of SBAs, such as DCA, is regulated by the gut microbiota and could promote hepatocarcinogenesis by activating the pathway of mammalian target of the rapamycin in hepatocytes (31). The prevalence of nonalcoholic fatty liver disease (NAFLD) is mainly increased by obesity and type 2 diabetes mellitus, and high-fat or high-cholesterol diets are closely linked to NAFLD-associated HCC, which animal experiments and clinical research have confirmed (32, 33). The pathological mechanism associated with gut microbiota dysbiosis involves an increase in the gut bacterial metabolite taurocholic acid and a decrease in 3-indolepropionic acid, as observed in the metabolomic profiling of the serum in high-fat/high-cholesterol diet-fed mice (34). The underlying molecular mechanisms involved in HCC progression include the gut microbial dysbiosis that can lead to enhanced serum taurocholic acid and reduced 3-indolepropionic acid levels that can promote cell propagation and lipid accretion, leading to NAFLD-associated HCC (34). In addition, obesity-associated tumorigenesis develops *via* NAFLD, continuing to NASH and eventually to HCC (35). The constituent of gram-positive bacterial cell walls, lipoteichoic acid, cooperates with DCA to promote NASH-associated HCC development in the tumor microenvironment *via* the microbiota-driven cyclo-oxygenase 2 pathway under the gut-liver axis (36). This implies that the abundance of the PBAs and SBAs, which is influenced by changes in the gut microbiota, shows significant associations between risk factors and effective strategies for the prevention of HCC.

The key metabolites produced by gut microbial fermentation of dietary fiber are short-chain fatty acids (SCFAs), which have a vital function in maintaining intestinal homeostasis. An epidemiological study of 125,455 participants from two cohorts in the USA suggested that the increased dietary fiber and whole grains intake was linked to lower HCC development risk (37). Treatment with SCFAs significantly decreased the HCC-positive mouse numbers and prevented the chronic liver disease progression to HCC (McBrearty et al., 2021). The molecular mechanism behind this was that SCFAs upregulated tumor suppressor disabled homolog 2 expression and partially blocked the activity of hepatitis B X antigen (HBx) (38, 39). These results were consistent with those from an *in vivo* model of HBx transgenic mice in which all the mice developed hepatitis and steatosis, which progressed to dysplastic nodules and noticeable HCC by 1 year (40). Propionate is a health-promoting SCFA that is produced by gut bacterial fermentation of indigestible fiber and is a principal metabolite that maintains intestinal homeostasis (41). Kobayashi et al. found that propionate enhanced the sensitivity of cisplatin and cisplatin-induced apoptosis of HCC cell lines through a GPR41-dependent pathway (42). The above findings demonstrate that SCFAs could be a possible treatment target to delay the development of HCC **(Table 1)**.

**Table 1:** Dysbiosis of bacterial metabolism and hepatocarcinogenesis.

Bacterial metabolites	Resource	Comparison	Outcomes	Mechanism of action	References
DCA	C57BL/6 (30-week-old mice)	Neonatal DMBA plus obesity-induced HCC model *vs*. oral antibiotic cocktail	DCA concentration decreased and HCC development suppressed	Enterohepatic circulation of DCA promoted hepatic stellate cell activation and induce obesity-associated HCC	(30)
	C57BL/6J (8-week-old mice)	Control standard diet *vs*. STHD-01 or STHD-01 plus antibiotics	STHD-01 contributed to process of NASH-associated HCC	Secondary bile acid DCA regulated by gut microbiota contributed to HCC *via* activation of mTOR signaling	(31)
TCA	C57BL/6 (8-week-old male mice)	Normal chow *vs*. high-fat/high-cholesterol diet	Alteration of TCA and IPA metabolites promoted hepatic lipid accretion and cell propagation	Cholesterol contributed to gut microbiota alteration, culminating in HCC development from NAFLD	(34)
IPA					
LTA + DCA	C57BL/6 (30-week-old mice)	Mice with normal diet *vs*. HFD	DCA administration promoted HCC development	LTA and DCA cooperatively promoted obesity-associated HCC development through gut microbiota	(36)
SCFAs	HBxTg mice (C57Bl/6 × DBA, 12 months old)	SCFA-treated *vs*. PBS fed HBxTg mice	SCFA treatment reduced number of HCC nodules	SCFA treatment promoted expression of DAB2 and depressed Ras pathway activity	(43)
TMAO	Fasting serum samples	671 PLC patients *vs*. 671 controls	TMAO higher serum levels connected with PLC risk increased	TMAO led to PLC development by decreasing the size of the total bile acid pool	(44)

TCA, taurocholic acid; DCA, deoxycholic acid; HCC, hepatocellular carcinoma; IPA, 3-indolepropionic acid; NAFLD, non-alcoholic fatty liver disease; NASH, nonalcoholic steatohepatitis; SCFAs, short-chain fatty acids; HFD, high-fat diet; PLC, primary liver cancer; LTA, lipoteichoic acid; TMAO, trimethylamine N-oxide.

A cross-sectional case-control study involving patients with primary liver cancer (n = 671) and 671 controls was conducted to examine the relationship between the levels of metabolites dependent on the gut microbiota, trimethylamine N-oxide and choline, and risk of primary liver cancer. Serum trimethylamine N-oxide level was positively related to primary liver cancer development, particularly in patients with lower levels of choline in the serum (44). Several studies have examined the concentration of ammonia during end-stage liver disease, including NASH and cirrhosis, and hyperammonia is linked with liver disease severity while scavenging ammonia prevents the progression of fibrosis in NAFLD (45, 46). Ammonia is generated by gram-negative anaerobes or gram-positive non-sporing anaerobes in the gut. It can enter the bloodstream and activate hepatic stellate cells (HSCs) and contribute to HCC occurrence and development (47).

## Probiotics in HCC

Probiotics are living microbes that confer beneficial effects to the host and help to maintain the stability of an organism as a whole (48). The well-known probiotic strains, e.g., *Lactobacillus* and *Bifidobacterium*, are involved in preserving gut microbiota homeostasis. Several beneficial strains for human health including *Faecalibacterium prausnitzii*, *Akkermansia muciniphila*, and other SCFA-producing bacteria require further investigation (48–51). Recent studies have revealed that various probiotic strains exert antiproliferative and proapoptotic effects on carcinoma cells and tumor-bearing or tumor-induced animal models (52, 53). The derangement of the gut flora has an important function in a variety of liver diseases, which includes HCC (54). Kumar et al. showed that probiotic fermented milk prepared with probiotic lactic acid bacteria *Lactobacillus rhamnosus* GG (LGG) and *Lactobacillus casei* strain Shirota could decrease the risk of HCC by regulating lower genotoxicity or oncogene expression and DNA damage levels (55). Furthermore, Prohep reduced subcutaneous HCC in a tumor model in mice, as there was a 40% reduction in tumor weight and size in comparison with that of the control group (56). Prohep is a novel probiotic blend that comprises three ingredients, LGG, heat-inactivated VSL#3, and viable *Escherichia coli* Nissle 1917. The possible mechanism of action of Prohep in preventing the development of HCC is the induction and emission of anti-inflammatory interleukin (IL)-10 and suppression of the differentiation of Th17 cells to regulate the gut microbiota and weaken tumor growth and angiogenesis (56). There have been several *in vitro* investigations of the effects of probiotics against fibrogenesis. Liver fibrosis is caused by an increase in extracellular interstitial protein levels, which leads to the cirrhosis and HCC development. Kanmani et al. reported that the probiotics (*Weissella cibaria*, *Lactobacillus brevis*, and *Lactiplantibacillus plantarum*) might reduce HSC activation and reduce collagen deposition in human LX-2 cell lines by modifying the signaling of transforming growth factor (TGF)-β/SMAD and inducing autophagy and apoptotic pathways (57). This study reported the beneficial role of probiotics in the inhibition of liver fibrogenesis and the prevention of liver cancer. Another study has shown the protective effects of LGG against patulin-induced apoptosis in HepG2 cells by regulating the levels of proapoptotic BCL2 proteins p53 upregulated modulator of apoptosis and BH3-interacting domain death agonist *in vitro* (58).

Several chemical-induced HCC mouse models have been used to investigate the effects of gut probiotics on healthy individuals. Diethylnitrosamine (DEN) is the most extensively used toxic chemical that induces human liver fibrosis and cirrhosis resulting in HCC development (59). Recent studies have reported that chronic DEN treatment of rats increases the number of cecal *E. coli* and *Atopobium* while inducing significant inhibition of the *Lactobacillus* spp., *Bifidobacterium* spp., *Enterococcus* spp., and *Bacteroides fragilis* groups. Probiotics alleviated hepatocarcinogenesis induced by DEN by re-establishing homeostasis of the gut and improving intestinal and hepatic inflammation. Administration of probiotics could inhibited liver cirrhosis progression to HCC through endogenous damage-associated molecular pattern and exogenous pathogen-associated molecular patterns inflammatory signaling pathways (60). Another study reported excessive *E. coli* growth in the guts of patients with HCC, which contributed to the hepatocarcinogenesis process (61). A rat model of DEN-induced hepatocarcinogenesis showed that probiotics could potentially be an effective treatment for HCC (62). Indeed, exopolysaccharides are highly viscous microorganism-produced and secreted carbohydrate polymers (63) that have immune, antitumor, and potent antioxidant properties. *Lactobacillus acidophilus* ATCC 4356 exopolysaccharides exhibit a preventive and therapeutic effect against DEN-induced HCC through the regulation of inflammatory biomarkers IL-17 and TGF-β1 and TLR2/STAT-3/P38-MAPK signaling pathways (62). This suggests that the potential mechanism of the effect of the gut microbiota on HCC includes changes in the intestinal microbiome composition that affects the function of hepatic immune cells. Several studies have shown that the gut microbiota modulates the immune system in patients with HCC by reducing CD8^+^ T cells (23), stimulating inflammatory response (64), and modulating immunosurveillance (22). Xie et al. explored the dynamic alterations in the microbiota of the gut in HCC development and progression using a mouse model of streptozotocin/HFD-induced NASH-HCC. Their work identified that the abundance of major bacterial species, including *Bacteroides* spp. and *Desulfovibrio* spp., were amplified in the group given streptozotocin/HFD, while the abundance of *Barnesiella* spp., *Odoribacter* spp., and *Paraprevotella* spp. was significantly decreased (65). The increased abundance of bacterial species correlated positively with lipopolysaccharide levels and the host pathophysiological features.

Clinical trials examining the connection between gut bacterial microbiota and HCC *via* the gut-liver axis are underway. Alterations in the populations of live bacteria in the intestines are linked to liver function, implying that changes in the gut microbiome serve as biomarkers for early detection of HCC **(Table 2)**. A recent study reported that the presence of qualitative and quantitative differences between the microbiota of the gut in patients with HCC related to NAFLD when compared with that of the healthy controls. The numbers of Bacteroidales, Bacteroidaceae, Bacteroides, Gemellaceae, and Enterococcaceae identified in the fecal samples from cirrhotic patients with HCC were increased, while there was a decline in the numbers of Bifidobacteriaceae, Verrucomicrobiaceae, Akkermansia, Adlercreutzia, and Dialister when compared to those in the controls (66). In patients with HCC, *Bifidobacterium* was depleted and its abundance was correlated inversely with the concentration of calprotectin, thereby showing a link with intestinal inflammation. The results suggested that systemic inflammation and the profile of the gut microbiota correlate significantly in NAFLD-related cirrhosis and contribute to HCC development. Interestingly, in a large cohort study from three regions in China with a total of 486 fecal samples from 135 healthy controls, 311 patients with hepatic tumor and 40 patients with cirrhosis were used to clarify how the gut microbiome might be used to noninvasively identify biomarkers of HCC (67). In the study, 419 samples underwent 16S rRNA sequencing with MiSeq to construct an early HCC diagnostic model. The results indicated that the diversity of the gut microbes was increased from cirrhosis to early HCC. There was a decrease in the abundance of bacteria that produce butyrate, such as Oscillibacter, Ruminococcus, Clostridium cluster IV, Faecalibacterium, and Coprococcus, while there was an increase in lipopolysaccharide-producing bacteria, including *Haemophilus* and *Klebsiella*, in early HCC when compared with those of the healthy controls (67). In addition, it has recently been demonstrated *via* high-throughput amplicon sequencing of the 16S rRNA gene that patients with primary HCC exhibited an increased degree of dysbiosis, calculated based on abundance of probiotic and harmful bacterial genera, in comparison to the healthy controls. The patients’ fecal samples showed that the relative abundance of *Desulfococcus*, *Enterobacter*, *Prevotella*, and *Veillonella* was enhanced at all stages, while the patients with primary HCC (stages I-III) had reduced *Cetobacterium* (68). In line with this, several studies have noted a correlation between intestinal dysbiosis and chronic liver disease (69, 70) and have proposed a novel integrated index degree for dysbiosis (*D_dys_*) as a tool for evaluating the discrepancies between the microbiota of the gut and the development of HCC.

**Table 2:** Summary of the probiotics associated with HCC.

Probiotic bacteria	Resource	Comparison	Outcomes	Mechanism of action	References
LGG and *Lactobacillus casei* strain Shirota	Male Wistar rats (4 weeks old)	AFB1 induced group *vs*. probiotic fermented milk with chlorophyllin-treated group	Probiotic lactic acid bacteria and chlorophyllin inhibited AFB1-induced HCC	Reduction in lower genotoxicity and oncogene express	(55)
LGG, viable *Escherichia coli* Nissle 1917, and heat-inactivated VSL#3	Male C57BL6/N mice (5-6 weeks old)	Control *vs*. cisplatin *vs*. ProTreat *vs*. ProPre group	Probiotics inhibited growth of liver tumor	Cytokine induction and suppressed Th17 cell differentiation in the gut	(56)
LAB, e.g., *Lactobacillus brevis*, *Lactiplantibacillus plantarum*, and *Weissella cibaria*	Human hepatic stellate cell line	LAB strains stimulation *vs*. control	LAB reduces HSCs activation	LAB mediates variation of the signaling pathways induced by TGF-β	(57)
LGG	Human hepatoma HepG2 cell	Control group *vs*. LGG alone group or LGG and Patulin group	Treatment of HepG2 cells with LGG inhibited patulin-induced toxicity	Regulation of BCL2 family members PUMA and BID mediated cell damage	(58)
*Lactobacillus acidophilus* ATCC 4356 EPSs	Adult male Swiss albino rats (180-220 g)	Control group *vs*. DEN group *vs*. EPSs group *vs*. EPSs + DEN *vs*. DEN + EPSs	EPS from *Lactobacillus acidophilus* ATCC 4356 against DEN induced HCC	Regulating the inflammation-associated P38-MAPK pathway	(62)
*Atopobium* spp. ↑, *Bacteroides vulgatus* ↑, *Bacteroides* spp. ↑, *Bacteroides uniformis* ↑, *Bacteroides acidifaciens* ↑, *Clostridium xylanolyticum* ↑, *Clostridium cocleatum* ↑, *Desulfovibrio* spp. ↑	Newborn male C57BL/6J mice	Normal diet-fed control mice *vs*. NASH-HCC model group injection of STZ and feeding with HFD	Gut microbiota changes during the HCC process	Possibly helpful bacteria correlated negatively with lipopolysaccharide (LPS)	(65)
*Parasutterella* spp. ↓, *Odoribacter* spp. ↓, *Bacteroides acidofaciens* ↓, *Moryella* spp. ↓, *Barnesiella* spp. ↓, *Lactobacillus intestinalis* ↓, *Paraprevotella* spp. ↓, *Akkermansia* spp. ↓					
*Atopobium* cluster ↑, *E. coli* ↑	Pathogen-free male Sprague-Dawley rats	Normal *vs*. DEN-induced HCC for 10 weeks	Disruption of gut homeostasis	Probiotics treatment prevented HCC development through intestinal flora balance	(60)
*Bifidobacterium* spp. ↓, *Lactobacillus* spp. ↓, *Enterococcus* spp. ↓, *B. fragilis* ↓					
*Bacteroidales* ↑, *Bacteroidaceae* ↑, *Bacteroides* ↑, *Gemellaceae* ↑, *Enterococcaceae* ↑	Patients with cirrhosis and HCC	Cirrhosis with HCC *vs*. no HCC	Alteration of the qualitative and/or quantitative disparities in the gut microbiota of patients with HCC	Gut microbiota with the link of inflammatory or immunological in patients	(66)
*Bifidobacteriaceae* ↓, *Verrucomicrobiaceae* ↓, *Akkermansia* ↓, *Adlercreutzia* ↓, *Dialister* ↓					
*Oscillibacter* ↓, *Ruminococcus* ↓, *Clostridium IV* ↓, *Faecalibacterium* ↓, *Coprococcus* ↓	Patients with HCC	Healthy controls *vs*. patients with early HCC	Decreased bacterial genera that produced butyrate and increased LPS-producing genera	Gut microbial alteration may have contributed to process of HCC	(67)
*Klebsiella* ↑, *Haemophilus* ↑					
*Desulfococcus* ↑, *Enterobacter* ↑, *Prevotella* ↑, *Veillonella* ↑	Primary HCC (stages I-III)	Different primary HCC stages *vs*. healthy controls	HCC patients with increased proinflammatory bacteria	Gut bacterial genera correlated with primary HCC development	(68)
*Cetobacterium* ↓					

LGG, Lactobacillus rhamnosus GG; LAB, lactic acid bacteria; AFB1, aflatoxin B1; HSCs, hepatic stellate cells; HCC, hepatocellular carcinoma; DEN, diethylnitrosamine; ↑ = upregulated; ↓ = downregulate.

## Antibiotics in HCC

Antibiotic treatments are related to decreased diversity of commensal bacteria, and the early use of antibiotics when treating malignancies is considered to be a disadvantage as it reduces the abundance of *Bifidobacteria*, *Akkermansia*, and *Ruminococcus* in the gut microbiota and increases that of the *Bacteroides* (71, 72). Clinical studies have also shown that antibiotic treatments are linked with a worse overall survival in patients with advanced HCC who received treatment with first-line sorafenib therapy. Antibiotics can cause changes in gut microbiota that influence reactions in systemic therapy (26). Prospective studies should focus on elucidating the mechanism behind the failure of the multitargeted tyrosine kinase inhibitors for HCC that caused by antibiotics which lead to gut microbiota dysbiosis. Moreover, immune checkpoint inhibitors (ICIs) that target programmed cell death-1 and its ligand are new promising therapies that extend the survival of patients with HCC. The humanized monoclonal antibodies target inhibitory receptors to provoke an antitumor immune response and modulate T-cell activation by interrupting the delivery of inhibitory signals (73). A retrospective cohort study investigated the impacts of antibiotics on the clinical trial results of ICIs for treating patients with HCC (74). Antibiotics against anaerobes were linked with a higher risk of HCC-related death during ICI therapy when compared with antibiotics against aerobes. In a retrospective study, 59 patients with advanced HCC from two academic centers were exposed to antibiotics while receiving ICI nivolumab treatment. Patients who received antibiotics had a shorter median overall survival than those who did not (*P* = 0.04). This suggests that patients with HCC receiving nivolumab have worse survival rates if they receive antibiotics, and that antibiotics should be used carefully during ICI treatments (75). In some other tumor types, multiple clinical trials have also demonstrated that early exposure to antibiotics was associated with poorer outcomes and reduced clinical benefits following ICI treatment, such as worse overall survival and increased disease refractory risk (76, 77). Future studies should include stool sampling, as well as biopsies and blood sampling, to focus on elucidating the mechanisms of the effect of antibiotics on the gut microbiota of patients with HCC treated with ICIs.

Some studies have assessed the effects of antibiotics in combination with anticancer therapies on the outcomes of patients with HCC. In an international multicenter cohort of 450 ICI recipients, early exposure to antibiotics was not disadvantageous to the therapeutic outcomes of immunotherapy in patients with HCC (78). Antibiotic exposure 30 days before or after ICI initiation during early immunotherapy showed no association with overall survival but was significantly associated with prolonged progression-free survival. The efficacy of ICI therapy, mainly anti-PD-1 monotherapy, in 414 patients from 11 centers, suggests that antibiotics (beta-lactams or quinolones) administered 30 days pre- or post-ICI initiation in HCC are linked with extended progression-free survival (79). It is recommend that patients with HCC should be given broad-spectrum antibiotics before surgery. Furthermore, Wang et al. used a meta-analysis to assess the influence of prophylactic antibiotic treatments on infections and complications in transarterially treated patients with HCC (80). They showed that antibiotic prophylaxis may not be routinely necessary for postoperative infectious complications in patients with HCC who have been treated with transarterial therapy. However, in patients with HCC who have undergone biliary tract disease or biliary reconstruction surgery and enterobiliary anastomosis, the prophylactic use of antibiotics could reduce the risk of infection. In a spontaneous HCC model using MYC transgenic mice, a cocktail treatment with three antibiotics was invented to remove gram-positive bacteria to provoke hepatic natural killer (NK) T cell accretion and prevent liver tumor growth (81). They also found that vancomycin or cefoperazone alone was sufficient to increase hepatic NKT cells, while the effect of neomycin was weakened. Abdel-Hamid et al. examined the anticancer activity of some macrolide antibiotics including clarithromycin, azithromycin, and erythromycin on HCC development both *in vitro* and *in vivo* (82). Treatment with clarithromycin inhibited the proliferation of HepG2 cells with an IC_50_ of 3.13 μg/mL and induced the apoptosis pathway to suppress HCC in a DEN-induced rat model. The basic experimental research should include the mechanisms of antibiotic-mediated intestinal dysbiosis and anticancer immune response drivers.

## FMT in HCC

FMT involves transferring healthy donor stool to the gut of a recipient with a physical illness (83). FMT is a safe and effective alternative to treat a recurrent *Clostridium difficile* infection (84). Multiple randomized clinical trials have confirmed that FMT had a success rate of nearly 92% when used as a second-line treatment of recurrent *Clostridium difficile* infection (85, 86). Preliminary studies have proposed that FMT is a potential treatment for a variety of other conditions, which include obesity, functional gastrointestinal disorders, inflammatory bowel disease, and metabolic syndromes (87). According to several randomized clinical trials, healthy donor FMT could possibly heal liver disease (88). FMT administered *via* an oral capsule was safe in patients with recurrent hepatic encephalopathy and cirrhosis (NCT03152188, Phase 1). There was increased abundance of Ruminococcaceae and Bifidobacteriaceae while that of Streptococcaceae and Veillonellaceae was decreased in the post-FMT microbiota, which is associated with improved duodenal mucosal diversity and hepatic encephalopathy (89). A characteristic of NAFLD is excess accumulation of fat in the hepatocytes and it is estimated to be present in 10-40% of adults worldwide (90). NAFLD is a risk factor of cirrhotic and HCC progression (91, 92). Craven et al. reported that patients with NAFLD demonstrating elevated permeability of the small intestinal at baseline reduced significantly 6 weeks after allogenic FMT (NCT02496390, Phase 1 and 2). However, there were no significant changes hepatic proton density fat fraction (allogeneic or autologous) or resistance to insulin (93). In most cases, the cause of HCC is the underlying liver damage due to chronic hepatitis, and in some developed countries, the mortality rate of HCC associated with cirrhosis is increasing (94). Previous researchers have evaluated the alterations in the gut microbiota using metagenomic analysis and found a decreased expression of the antibiotic resistance gene in cirrhosis patients who underwent FMT *via* capsule or enema. These genes could be potential targets with healthy donor FMT (NCT03152188, Phase 1) (95). Moreover, many clinical trials have investigated the preliminary benefits of FMT in various liver diseases, such as: the effect of consecutive FMT on NAFLD with 20 participants through a randomized-controlled trial (NCT04465032, Phase 4); FMT using stool from five lean donors to determine the efficacy and safety for patients with NASH through single group assignments of interventional clinical trial (NCT02469272, Phase 1); and FMT versus standard therapy in NASH related cirrhosis with 112 participants through a randomized control trial (NCT02721264) **(Table 3)**.

**Table 3:** Registered clinical trials and animal studies using FMTs to treat liver disease.

Condition or disease	Enrollment	Phase	Outcomes	NCT number	References
Hepatic encephalopathy and cirrhosis	20 participants	Phase 1	Single administration of FMT enema using a rationally selected donor *via* OpenBiome is safe	NCT03152188	(89)
			Reduction in expression of the antibiotic resistance gene after FMT (capsule or enema) in cirrhosis patients		(95)
NAFLD	21 participants	Phase 1 and 2	FMT from lean healthy donors improved intestinal permeability at 6 weeks in patients with NAFLD	NCT02496390	(93)
Steatohepatitis induced by HFD	Three groups (12 mice per group)	—	FMT attenuated the steatohepatitis induced by HFD through the gut microbiota	—	(96)
Patients with NAFLD	20 participants	Phase 4	—	NCT04465032	—
Patients with NASH	5 participants	Phase 1	—	NCT02469272	—
Patients with NASH-related cirrhosis	112 participants	—	—	NCT02721264	—

FMT, fecal microbiota transplant; HFD, high-fat diet; NASH, nonalcoholic steatohepatitis; NAFLD, non-alcoholic fatty liver disease.

Data from animal studies have suggested that FMT alleviates steatohepatitis induced by HFD and has a beneficial outcome because of its alterations to the composition of the gut microbiota (96). In male C57BL/6 mice, FMT reverses gut microbiota dysbiosis and regulates SCFA levels by increasing the butyrate concentration in the cecum to correct the ratio of T-cell subsets that are beneficial to immunological balance. Bacteroidetes abundance was decreased whereas that of Firmicutes was increased after FMT when related with the HFD group (96). The direct effects of FMT on HCC growth and development have not been studied yet. The possible mechanism is that FMT regulates intestinal flora disorders and reduces cytotoxin production to inhibit the incidence of HCC in patients with hepatitis B cirrhosis (97). In addition, FMT can modulate the gut microbiome to abrogate ICI-associated colitis and toxicity (98), which implying the other mechanisms such as immune microenvironment could be involved in the effect of FMT on HCC. Thus, further investigations are needed to prove the safety of FMT in patients with HCC.

## Microbiome and CCA

After HCC, CCA is the most common malignant tumor and epithelial cell malignancy that accounts for almost 15% of all primary liver cancers. More than two-thirds of patients with CCA are diagnosed at an advanced disease stage with poor prognosis, as there is an absence of obvious symptoms to facilitate earlier diagnosis (99). Based on the anatomical sites of origin, CCA is categorized into three subtypes: intrahepatic, perihilar, and distal (100). Microbiota dysbiosis is closely linked with CCA progression from precancerous diseases (101). Gut microbiota can be used as a potential biomarker for CCA. In a case-control study, Zhang et al. performed high-throughput sequencing of the 16S rRNA from the fecal samples from 53 patients with CCA, 47 patients with cholelithiasis, and 40 healthy controls, and proposed a predictive model based on four enriched genera (*Bacteroides*, *Muribaculaceae_unclassified*, *Muribaculum*, and *Alistipes*) for the better diagnosis of CCA (102). Similarly, a classification model consisting of eight genera was constructed to classify CCA, HCC, and healthy controls (103).

In healthy people, the gut microbiota is ecologically balanced and meanwhile the link between gut microbiota and the pathogenesis of CCA is attracting more attention (104, 105). Biliary obstruction caused by CCA leads to cholestasis, which provides a culture medium for bacteria. Decreased bile secretion, low immunity of patients with CCA, and surgical trauma can promote overgrowth of the gut microbiota, which can result in the overgrowth of small intestinal bacteria, increased abundance of pathogenic bacteria, and change in microbiota abundance (105, 106). The overgrowth of gut microbiota can damage the intestinal mucosal barrier, which can lead to increased permeability of the intestinal mucosa; thus, a large amount of endotoxin and pathogenic bacteria permeate into the blood, resulting in intestinal endotoxemia and movement of the microbiota (107). This process leads to the inflammatory mediator release such as IL-1, TNF-α, and IL-6 which induces a series of inflammatory immune responses (108, 109). Moreover, long-term low-grade chronic inflammation is related to cancer incidence and development. At the same time, the gut microbiota can enter the biliary tract through the blood, lymph, or in a retrograde manner leading to a biliary infection (110).

Bile duct stones are a risk factor for intrahepatic CCA development, as a systematic review reported that there was a strong correlation between hepatolithiasis and intrahepatic CCA (111). Liver flukes are linked to bile duct infection and proinflammatory cytokine production which was consistent with the inflammatory phenotype in CCA development (112–114). Chng et al. reported that the *Stenotrophomonas* species abundance was significantly increased in tumor tissues of non-fluke-related CCA (115). The pathogenesis of CCA may involve the microbial community, as reported by Saab et al., who found that *Streptococcus*, *Enterococcus*, *Klebsiella*, *Bacteroides*, and *Pyramidobacter* were the most abundant in the bile of patients with extrahepatic CCA. The bile of patients with CCA had lower *Firmicutes* abundance and higher *Bacteroidetes* abundance than those of benign biliary pathology (116). The microbiota of the bile from patients with distal CCA was highly enriched with *Proteobacteria*, *Firmicutes*, *Bacteroidetes*, and *Actinobacteria*. Moreover, the abundance of phyla *Nitrospirae*, *Gemmatimonadetes*, *Latescibacteria*, *Chloroflexi*, and *Planctomycetes* were significantly increased in patients with distal CCA when compared with patients with cholelithiasis (117). However, when compared with HCC, liver cirrhosis, and healthy controls, Jia et al. reported that patients with intrahepatic CCA demonstrated the highest levels of species richness, with four highly enriched genera in the gut microbiota, *Lactobacillus*, *Actinomyces*, *Peptostreptococcaceae*, and *Alloscardovia* (109). The *Ruminococcaceae* family is also enriched in patients with intrahepatic CCA who have vascular invasion. Patients with intrahepatic CCA have high plasma-stool ratios of tauroursodeoxycholic acid and glycoursodeoxycholic acid, which can be used as biomarkers to distinguish intrahepatic CCA from HCC (109). These studies indicate the microbiome diversity in patients with CCA and the heterogeneity among individuals.

A potential risk in CCA development is choledocholithiasis (CDL), which is a consequence of bacterial infection. Dangtakot et al. performed a sequencing study of the 16S rRNA gene, including samples of bile from 30 patients with CDL and 30 with CCA. Results showed higher abundance of *Enterobacter*, *Pseudomonas*, and *Stenotrophomonas* species in CCA when related with CDL (*P* < 0.05), while the *Pyramidobacter*, *Cetobacterium*, and *Streptococcus* species were less abundant in CCA when compared with CDL (*P* < 0.05) (118). The chronic liver disease, primary sclerosing cholangitis is accompanied by inflammation and scarring and is an important risk factor in CCA (119). It was recently reported that the gut microbiome directs hepatocytes through myeloid cell accumulation to control CCA; and when treated with neomycin to eliminate gram-negative gut bacteria, CCA inhibition could be achieved in the bile duct ligation-modeled mice (120). The bacteria that commonly exist in the environment although not in humans, such as Nesterenkonia, Methylophilaceae, and Mesorhizobium, were found in the microbiota of patients with extrahepatic CCA and benign biliary pathology. In a multicenter case-control study, the abundance of *Actinomyces*, *Novosphingobium*, *Prevotella*, and *Fusobacterium* were significantly increased in extrahepatic CCA, while that of *Nesterenkonia* and *Rothia* were reduced (121). Serra et al. found in bile samples that *Pseudomonas* was a significant positive prognosticator for CCA (122), and Lee et al. reported that *Pseudomonas* was a significant negative biomarker for CCA using plasma samples (123). These contrasting results might be due to the differences in sample types and study objects. When compared with bile samples, the blood microbiome of patients with CCA requires further investigation to explore its conformation and mechanisms.

The functional mechanisms of the microbiota of the gut in CCA have been inadequately studied. The gut microbiome directs hepatocytes and promotes CXCL1 expression in a TLR4-dependent manner to recruit myeloid-derived suppressor cells and promote CCA (120). Using large-scale proteogenomics, Dong et al. yielded insights into intrahepatic CCA etiology and taxonomy. They identified four intrahepatic CCA subgroups that were associated with different molecular and clinical features and selected HKDC1 and SLC16A3 as prognostic biomarkers (124).

## Microbiome and GBC

GBC is a common malignant tumor with poor prognosis and almost all types are adenocarcinomas that are usually diagnosed at an advanced stage (125). GBC is recognized as a highly lethal disease due to a lack of predictive radiological features and accurate identification, which leads to only 10% of patients presenting at a stage suitable for surgical resection (126). Multiple investigations have demonstrated that bacterial infections are related to higher GBC risk. Choi et al. reported the bacterial species present in the bile microbiome, through metagenomic sequencing from patients with normal gallbladder, chronic cholecystitis, and GBC. They showed significantly increased *Escherichia*, *Enterobacter*, and *Klebsiella* in GBC samples when compared with that in normal bile samples. *Klebsiella* abundance between patients with chronic cholecystitis and GBC was significantly different (127), which indicates a link between microbiological disorders and GB carcinogenesis. Another study clarified the microbial communities between patients with GBC and cholelithiasis using next-generation-based 16S rRNA sequencing. *Enterobacter* sp. and *Fusobacterium nucleatum* were predominant and identified in the GBC samples, while *Salmonella* sp. and *Enterococcus gallinarum* were prevalent in the bile of cholelithiasis patients (128). A chronic *Salmonella* carrier state is related to GB carcinogenesis, especially in areas with high typhoid endemicity (129). This is consistent with the finding that *Salmonella typhi* in the gallbladder of gallstone patients may be a cause for the development of GBC (130). A recent study investigated if *Salmonella* infection is a risk factor for GBC in genetically susceptible mice through the regulation of AKT and MAPK pathways during infection (131). The results indicated that *Salmonella enterica* can promote carcinogenesis and is a causative agent of GBC. Thus, eradicating bacterial infection *via* immunotherapy, anti-inflammatory therapy, and hygienic practices might help in reducing the incidence of GBC.

## Discussion

**Figure 1:**
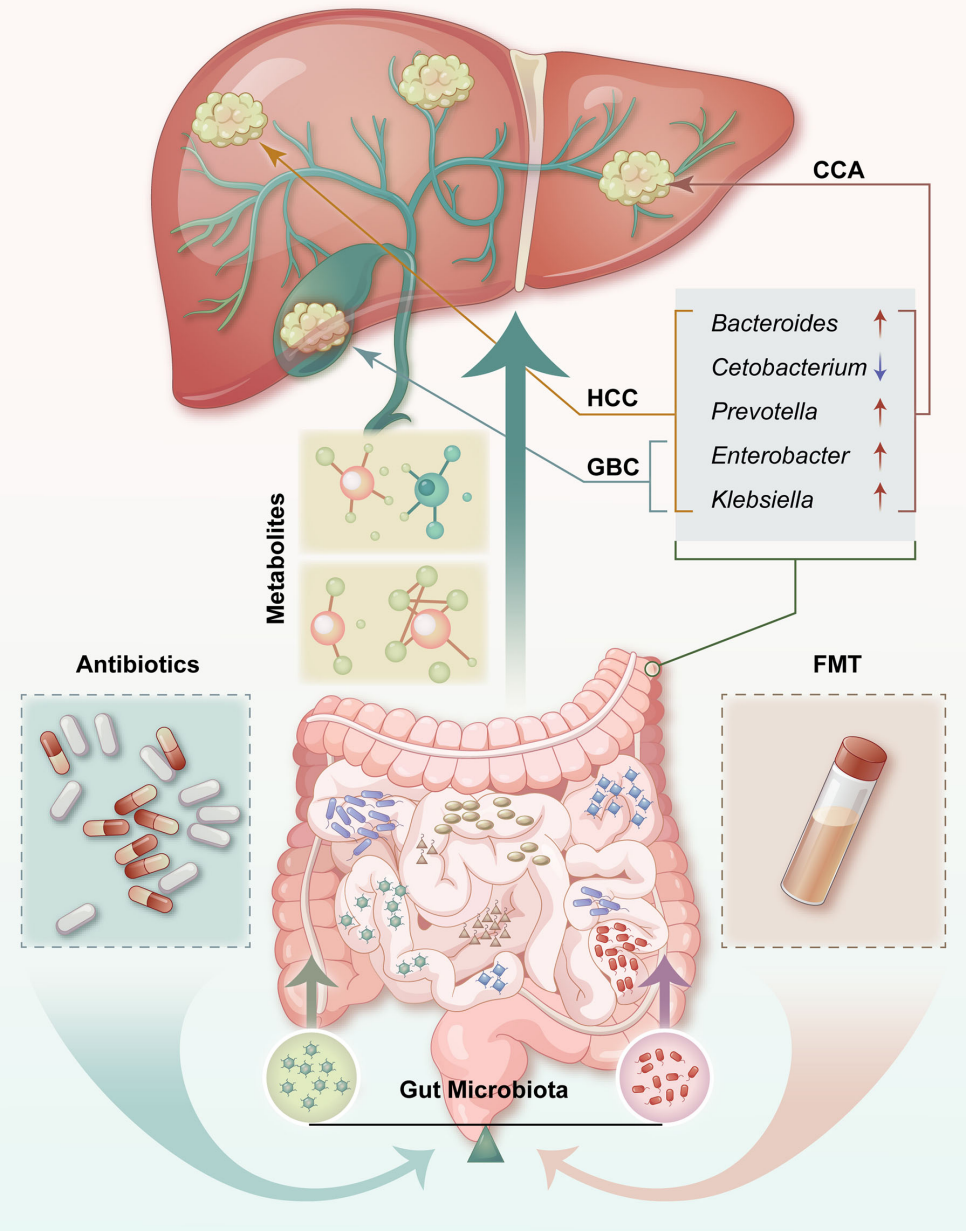
Contributions of the microbiota of the gut to hepatobiliary tumors. There is an anatomical and physiological connection between the liver and the intestinal tract. Potential effective treatments or preventive strategies for HCC, including probiotics, antibiotics, and FMT, may be cost-effective. HCC, hepatocellular carcinoma; CCA, cholangiocarcinoma; GBC, gallbladder cancer; FMT, fecal microbiota transplant; ↑ = upregulated; ↓ = downregulated.

There is growing proof for the profound impact of gut homeostasis on HCC development, and consequently, gut microflora may embody a novel target for prevention of primary HCC. Due to its anatomical position, the liver is subjected to enteric bacterial components, meaning intestinal bacterial products could spread to the liver through the portal vein (132). Many have examined the alterations that occur in the microbiomes of patients with CCA and GBC, and it was found that dysbiosis of the microbiome could promote tumorigenesis and GBC and CCA development (101, 128). In this review, we have summarized the data regarding the bacteria that are altered in the microbiome, such as *Enterobacter* and *Klebsiella* and the gram-negative bacteria that co-occur in cirrhotic patients with HCC using samples from patients with GBC (**Figure 1**). There was a significant increase in *Enterobacter* in GBC samples when compared with the normal bile samples, and its abundance was enhanced in the fecal samples of patients with HCC at all disease stages (68, 127). It has been reported that *Enterobacter* is related to a proinflammatory response (133). The above phenotype was consistent with an investigation, which found that inflammation is central to GBC development and that reducing inflammation-related pathways was associated with increased survival during GBC outcomes (134). Inflammation is a critical component of HCC progression and anti-inflammatory drugs are reported to enhance the prognosis of patients with HCC (135). There was increased *Bacteroides*, *Prevotella*, *Klebsiella*, and *Enterobacter* in the stool samples from cirrhotic patients with HCC and the biliary microbiota of patients with CCA as compared with the control groups (66, 116). Gram-negative *Bacteroides* are correlated with chronic intestinal inflammation and tissue injury, which are colorectal cancer risk factors (136). Chronic and unresolved inflammation is regarded as the main carcinogenic mechanism promoting GBC development (137), as it provides a possible underlying mechanism, in which *Bacteroides* have essential roles in HCC and GBC incidence through gut-derived inflammation. In this context, human bacteria, such as *Enterobacter* and *Bacteroides*, coexist with their host and facilitate multiple types of carcinogenesis. Other studies have also reported that augmented *Actinomyces* abundance in the bile ducts of patients with extrahepatic CCA may have originated from the intestine (109).

Probiotics are used to modulate the intestinal microflora balance and confer health benefits to the host. LGG is a widely used probiotic strain that has been evaluated for the treatment of diarrhea (138), antiproliferative effects on the cells of colon adenocarcinoma (139), suppressive effects on inflammation during *Helicobacter* infection in gastric adenocarcinoma cells (140), and high-fructose diet-induced NAFLD (141). In the present review, we found that probiotic LGG can decrease the risk of HCC through multiple pathways, including DNA damage (55), inflammatory and cell differentiation (56), and human hepatoma cell apoptotic regulation (58). Probiotics interact with the host and the microbiome *via* molecular effectors as metabolic products through the gastrointestinal microenvironment and mediate health benefits (142). Intestinal dysbiosis influences HCC development partly *via* microbial and gut metabolites and products conveyed through the portal vein to the liver, which could be a key regulator of tumorigenicity and HCC aggressiveness (143). This was consistent with a previous study that explored the molecular mechanisms of gut dysbiosis on gut barrier and inflammation in relation to carcinogenesis, such as immune responses, toxic metabolites, and signaling pathway intervention (144). The metabolic byproduct of intestinal bacteria, DCA, could contribute to HCC *via* multiple signaling pathways such as the activation of mammalian targets of rapamycin signaling or the gut microbiota-driven cyclo-oxygenase 2 pathway (31, 36). It was recently reported that DCA induced human HSC malignant behavior of HCC by inducing senescence-associated secretory phenotype factors, e.g., TGF-β and IL-8 (145). The above investigations suggest that multiple actions of gut metabolites could control cancer growth and progression in different HCC models.

In conclusion, we have summarized the role of microbiota of the gut in hepatobiliary tumor incidence and development, including HCC, CCA, and GBC. The potential effectiveness of the treatments and representative novel strategies, including probiotics, antibiotics, and FMT, focus on fecal microbiome modulation, which provides alternative therapeutic methods for HCC. There are still many questions regarding microbiota function in the occurrence of HCC that remain unclear. There are limitations to the gut microbiome as a biomarker or therapeutic target for hepatobiliary tumors, since the gut microbiome is easily disturbed by several confounding factors, such as diet, medication (especially probiotics and antibiotics), mental health, and environmental factors, which make this type of biomarker not robust or reliable. In addition, interruption of the balance of the gut microbiome could lead to a series of problems, thus a global view should be taken before using the gut microbiome as a therapeutic target. The gut microbiome and hepatobiliary tumors are not directly linked spatially, which means that the gut microbiome does not reflect hepatobiliary tumors straightforwardly. The gut microbiome is involved in hepatobiliary tumor development through complex mechanisms, and there is an urgent need for more laboratory investigations and extensive human clinical trials to assess the gut microbiota abundance and screen for beneficial strains.

## Author Contributions

YW designed the content of the review with input from all the co-authors. HQ and BY drafted the manuscript. YW and WH revised the manuscript. All authors contributed to the article and approved the submitted version.

## Funding

This study was supported by grants from The National Natural Science Foundation of China (42125707, 41931291, 81902960), the Non-profit Central Research Institute Fund of Chinese Academy of Medical Sciences (2019PT310027), the Chinese Academy of Medical Sciences Innovation Fund for Medical Sciences (2019-I2M-1-003, 2021-1-I2M-018, 2021-RC310-018).

## Conflict of Interest

The authors declare that the research was conducted in the absence of any commercial or financial relationships that could be construed as a potential conflict of interest.

## Publisher’s Note

All claims expressed in this article are solely those of the authors and do not necessarily represent those of their affiliated organizations, or those of the publisher, the editors and the reviewers. Any product that may be evaluated in this article, or claim that may be made by its manufacturer, is not guaranteed or endorsed by the publisher.

## Abbreviations

HCC, hepatocellular carcinoma; FMT, fecal microbiota transplantation; CCA, cholangiocarcinoma; GBC, gallbladder cancer; PBA, primary bile acids; SBA, secondary bile acid; DCA, deoxycholic acid; NAFLD, nonalcoholic fatty liver disease; NASH, nonalcoholic steatohepatitis; SCFA, short-chain fatty acid; HSC, hepatic stellate cell; LGG, *Lactobacillus rhamnosus* GG; TGF, forming growth factor; DEN, diethylnitrosamine; ICI, immune checkpoint inhibitors; CDL, choledocholithiasis.
